# Burning mouth syndrome in Parkinson’s disease: dopamine as cure or cause?

**DOI:** 10.1007/s10194-012-0421-1

**Published:** 2012-02-10

**Authors:** Elizabeth A. Coon, Ruple S. Laughlin

**Affiliations:** Neuromuscular Division, Department of Neurology, Mayo Clinic, Rochester, MN 55905 USA

**Keywords:** Parkinson’s disease, Burning mouth syndrome, Carbidopa/levodopa, Dopamine, Pain

## Abstract

Burning mouth syndrome has been reported as being more common in Parkinson’s disease patients than the general population. While the pathophysiology is unclear, decreased dopamine levels and dopamine dysregulation are hypothesized to play a role. We report a patient with Parkinson’s disease who developed burning mouth syndrome with carbidopa/levodopa. Our patient had resolution of burning mouth symptoms when carbidopa/levodopa was replaced with a dopamine agonist. Based on our patient’s clinical course, in conjunction with earlier studies assessing the relationship between burning mouth syndrome and Parkinson’s disease, we discuss a potential role for dopamine in burning mouth syndrome in Parkinson’s disease.

## Introduction

Burning mouth syndrome is characterized by a painful, intraoral burning sensation that lacks physical or laboratory findings. In the general population, postmenopausal women and the elderly are most often affected, with a prevalence ranging from 3.7 to 18% [1]. Burning mouth syndrome may be more common in Parkinson’s disease with one study reporting a 24% prevalence in Parkinson’s disease patients [2]. All of these patients developed burning mouth symptoms after the diagnosis of Parkinson’s disease and 96% were taking levodopa. However, this study was from a selected population in Northern Ireland and limited by size with only 115 valid respondents [2].

Diminished endogenous dopamine and dysregulation of dopaminergic receptors in the nigrostriatal pathway has been implicated as one pathophysiologic mechanism for primary burning mouth syndrome [3, 4]. We report a Parkinson’s disease patient who developed burning mouth syndrome after starting carbidopa/levodopa and had improvement of her symptoms after discontinuation of carbidopa/levodopa and resolution upon initiation of a dopamine agonist. Based on our case and findings from earlier studies, we discuss potential mechanisms of burning mouth syndrome in Parkinson’s disease.

## Case presentation

A 65-year-old right-handed woman was diagnosed with akinetic-rigid Parkinson’s disease after a 4-year history of progressive right arm stiffness associated with musculoskeletal pain. She had been maintained only on a low dose of pramipexole (0.25 mg three times daily) due to feelings of anxiety at higher doses. On initial neurologic examination, she demonstrated hypomimia and hypokinetic dysarthria. She had cogwheel rigidity and slowed rapid alternating movements with the right-upper extremity and a bradykinetic gait with absent right arm swing. Based on the patient’s reluctance to escalate the dose of pramipexole, it was tapered off and replaced with carbidopa/levodopa 25/100 mg tablets titrated to a dose of 1.5 tablets three times per day. On this regimen she experienced marked improvement in her Parkinson’s disease symptoms.

Six weeks after starting carbidopa/levodopa, the patient began experiencing burning, “peeling” sensations involving her tongue, cheeks and palate with associated dysgeusia. The pain typically worsened throughout the day and became severe enough to prompt an urgent care visit where she was prescribed a course of oral nystatin with no relief. Oral and dental examinations were normal and she experimented with different toothbrushes and toothpastes, ice cubes, gum and hard candies with avoidance of spices, mint and citrus products to no avail. Her mouth symptoms intensified after she was prescribed additional carbidopa for nausea associated with levodopa doses, leading to discontinuation of the additional carbidopa. She was evaluated by otolaryngology and dermatology and had a negative laboratory evaluation including complete blood count, vitamin B1, B2, B6 and B12, magnesium, zinc, folate, ferritin, thyroid stimulating hormone and anti-nuclear antibody. She was diagnosed with burning mouth syndrome and prescribed clonazepam 0.5 mg tabs to dissolve in the mouth which offered no relief. Due to the correlation of symptom onset with initiation of carbidopa/levodopa, this medication was discontinued. Over the next 2 weeks her symptoms improved with only residual dysesthesia affecting the tip of her tongue. Unfortunately, her Parkinson’s disease symptoms concomitantly worsened with return of her right upper limb rigidity and pain and new stiffness involving her left upper limb. Pramipexole was re-initiated and titrated to a dose of 1.5 mg orally three times daily which led to the improvement of her Parkinson’s disease symptoms similar to that achieved with carbidopa/levodopa and she had complete resolution of her burning mouth symptoms.

## Discussion

Carbidopa/levodopa was considered causative for burning mouth syndrome in our patient as symptom onset and severity correlated directly with its administration and titration. The response to dopaminergic therapy in our patient is unusual as central pain syndromes in Parkinson’s disease, such as oral and genital pain, are more likely to respond to dopaminergic therapy than other types of pain [5, 6]. Central and peripheral pain in Parkinson’s disease are recognized as distinct processes; the origin of central pain is not clearly understood while peripheral pain appears to be from abnormal nociceptive processing [6]. Of interest, our patient also exhibited musculoskeletal limb pain associated with Parkinson’s disease (on the affected side) which responded to levodopa therapy. This is consistent with previous reports in Parkinson’s disease where pain was predominantly a factor in the ‘off’ or dopamine deficient state [6]. Our case suggests that the mechanisms underlying burning mouth syndrome in Parkinson’s disease may differ from other types of pain in Parkinson’s disease.

Burning mouth syndrome has also been analogized to restless leg syndrome, a syndrome related to dopaminergic dysfunction [7]. In restless leg syndrome, high doses of dopamine may paradoxically lead to worsening and augmentation of symptoms after initial benefit [8]. One hypothesis is that overstimulation of pro-nociceptive D1 receptors compared to anti-nociceptive D2 receptors leads to pain. Studies supporting this have shown reduced D2 receptors, but not D1 receptors in hyperdopaminergic states [8–10]. In animal models, sensitivity of dopaminergic receptors has been augmented by levodopa, with a nociceptive effect that was depressed by blockage of D1 receptors [10].

Studies in primary burning mouth syndrome have shown dysfunction of the nigrostriatal dopaminergic pathway [1, 3, 4]. Positron emission tomography studies of burning mouth syndrome patients without Parkinson’s disease indicate low levels of endogenous dopamine in nigrostriatal neurons compared to controls [3], and demonstrate alterations in the binding of D1 and D2 receptors in the putamen [4]. This is intriguing as increasing central dopamine levels with additional carbidopa led to dramatic worsening in our patient’s burning mouth symptoms. Treatment was achieved by withdrawing excess dopamine and substituting pramipexole, a D2 and D3 dopamine receptor agonist.

While caution is advised in interpreting an individual case, burning mouth syndrome in our patient with Parkinson’s disease could be explained by dysregulation of dopamine receptors in an endogenous hypodopaminergic state. In the presence of excess dopamine, exaggerated D1 compared to D2 receptor activity may have caused burning mouth symptoms. Pramipexole has been reported as a therapy in primary burning mouth syndrome, and success in our patient supports its use as a treatment choice for burning mouth syndrome in Parkinson’s disease patients [7].

*Ethical statement* High standard of ethics according to the WMA Declaration of Helsinki was applied in all investigations and clinical work described in this manuscript.
